# Environmental Exposure Context and Advanced Stage at Diagnosis in Thyroid Cancer: A Population-Based SEER Analysis

**DOI:** 10.3390/cancers18162679

**Published:** 2026-08-19

**Authors:** Jingjing Tong, Sarat Chandra Gupta Surampalli, Srinidhi Santhosh Kumar, Naga Venkata Sriram Golli

**Affiliations:** Department of Data Analytics and Statistics, University of North Texas, Denton, TX 76203, USA

**Keywords:** thyroid cancer, SEER, advanced stage, environmental exposure, endocrine disruptors, feature importance, machine learning

## Abstract

Background: Environmental exposures are biologically plausible contributors to thyroid cancer outcomes, but population-based evidence is heterogeneous and often focuses on incidence rather than stage at diagnosis. Objective: This study aimed to evaluate whether ecological state-year environmental indicators add predictive information for advanced-stage thyroid cancer at diagnosis in a large SEER-based cohort (U.S. National Cancer Institute’s Surveillance, Epidemiology, and End Results Program). Methods: We conducted a retrospective registry-linked analysis of 368,726 SEER thyroid cancer cases with valid combined summary stage. Advanced stage was defined as regional or distant disease and occurred in 118,509 cases (32.14%). Environmental variables were linked as state-year indicators using five-year moving averages from the years preceding diagnosis. Regularized logistic regression, gradient boosting, and Extra Trees models evaluated incremental predictive performance. Results: Adding environmental variables to demographic, socioeconomic, histology, and laterality predictors produced small random-split improvements across model families. However, after SEER registry was added before environmental variables, the remaining environmental increment was minimal. Registry-group holdout validation did not support improved geographic generalizability from the environmental feature set. Conclusions: State-year environmental indicators carried limited predictive information for advanced thyroid cancer stage, and much of this information overlapped with registry and geographic structure. These findings clarify both the potential and limitations of ecological environmental linkage in SEER-based prediction studies.

## Simple Summary

This study examined whether environmental context adds useful information for predicting advanced stage at diagnosis among patients with thyroid cancer in a large U.S. cancer registry cohort. We linked SEER thyroid cancer records with state-year environmental indicators summarized over the five years preceding diagnosis. Environmental variables slightly improved prediction when added to demographic, socioeconomic, and tumor-related features, but much of this information overlapped with SEER registry and geographic structure. The findings support cautious use of environmental context in population-based prediction models and emphasize that these state-level indicators should not be interpreted as individual exposure measurements or causal effects.

## 1. Introduction

Thyroid cancer is one of the most commonly diagnosed endocrine malignancies, and its incidence has changed substantially over recent decades. Increased detection of small, low-risk tumors has contributed to these trends, but thyroid cancer patterns also vary by geography, race/ethnicity, sex, socioeconomic context, and time period [1,2,3,4,5]. Advanced stage at diagnosis remains clinically meaningful because it reflects more severe disease presentation and is less directly equivalent to increased detection of low-risk tumors. Understanding contextual predictors of advanced stage may therefore provide insight into population-level patterns of thyroid cancer severity.

Environmental exposures have long been considered plausible contributors to thyroid disease and thyroid cancer. The thyroid gland is sensitive to hormonal regulation, iodine metabolism, radiation-related exposures, and chemicals that may disrupt endocrine signaling [6,7,8]. Prior studies have examined environmental exposures, air pollution, endocrine-disrupting chemicals, and radiation-related factors in relation to thyroid cancer or thyroid-related outcomes, but findings have been mixed [7,9,10,11,12,13,14,15]. Some studies report positive associations, whereas others report weak, null, or inconsistent findings. These differences may reflect variation in exposure measurement, geographic setting, sample size, outcome definition, tumor subtype, and overlap with socioeconomic and healthcare-access factors.

Moreover, several limitations in the existing literature make additional population-based analysis necessary. Many environmental studies of thyroid cancer have focused on incidence rather than advanced stage at diagnosis, even though stage provides a clinically meaningful measure of disease presentation. Other studies have examined single exposure classes, local or regional populations, or relatively small datasets, which can limit generalizability and make it difficult to compare findings across environmental domains [3,16,17,18]. Because environmental exposures are also spatially structured, studies must consider how exposure measures overlap with geography, socioeconomic conditions, and screening patterns.

Our study addresses these gaps using a large SEER-based thyroid cancer cohort with valid summary stage. We evaluated whether state-year environmental exposure attributes contributed predictive information for advanced stage at diagnosis when considered alongside demographic, socioeconomic, and tumor-related variables. The primary goal was prediction and incremental information assessment rather than causal estimation. Because the exposure measures were ecological indicators linked at state-year levels, the analysis was designed to clarify whether these contextual variables improved model performance and how their importance changed after accounting for SEER registry and geographic structure.

The remainder of this article is organized as follows. The Literature Review summarizes prior evidence on thyroid cancer trends, environmental and endocrine-disruptor risk factors, geography and socioeconomic context, and SEER-based research on thyroid cancer. The Section 3 describes the SEER-based cohort construction, outcome definition, predictor variables, environmental exposure-context measures, model comparison strategy, feature-importance approach, and sensitivity analyses. The Section 4 presents descriptive cohort findings, model performance comparisons, registry-adjusted environmental feature importance, and sensitivity analyses. The Section 5 interprets the findings in relation to the prior literature, emphasizes the ecological and geography-sensitive nature of the environmental signal, and outlines the strengths, limitations, and implications for future research.

## 2. Literature Review

### 2.1. Thyroid Cancer Trends and the Importance of Advanced Stage

Thyroid cancer incidence has increased substantially over recent decades, with marked variation across countries, regions, sex, age, race/ethnicity, and socioeconomic groups. Increased detection of small papillary thyroid cancers, wider use of diagnostic imaging, and changes in pathologic classification have contributed to these trends, and the problem of overdiagnosis is well recognized [1,2]. At the same time, geographic variation and changes in larger or clinically meaningful tumors suggest that thyroid cancer epidemiology cannot be explained only by detection intensity [3,4,5]. Population-level context therefore remains important when interpreting thyroid cancer patterns. Advanced stage at diagnosis is an important outcome for environmental and population-based thyroid cancer research because it reflects more severe disease at the time cancer is detected. Studies of thyroid cancer incidence can be strongly influenced by screening intensity, diagnostic access, and the detection of small, low-risk tumors. In contrast, stage at diagnosis provides information about the clinical severity of disease presentation.

### 2.2. Environmental and Endocrine-Disruptor Risk Factors

The thyroid gland is biologically plausible as a target for environmental attributes, particularly the endocrine-disrupting exposures, because thyroid function depends on regulated hormone synthesis, iodine metabolism, and hypothalamic–pituitary–thyroid axis signaling. Reviews have identified exposure classes of potential relevance to thyroid cancer or thyroid disease, including ionizing radiation, air pollution, industrial chemicals, pesticides, heavy metals, dioxins, polychlorinated biphenyls, per- and polyfluoroalkyl substances, and flame retardants [6,7,8,12]. These exposures may affect thyroid biology through endocrine disruption, oxidative stress, inflammation, altered iodine handling, or broader immune and metabolic pathways.

Despite biologic plausibility, epidemiologic evidence remains heterogeneous. Associations vary by exposure class, timing of exposure, tumor subtype, sex, geography, study design, and exposure measurement method. Some studies report positive associations between selected environmental exposures and thyroid cancer incidence or tumor characteristics, while others find weak, null, or inconsistent findings [7,9,10,11,12,13,14,19,20,21]. This variability argues against treating any single environmental feature as an isolated causal factor without careful adjustment for social, clinical, temporal, and geographic structure.

### 2.3. Geography and Socioeconomic Context

Environmental exposures are spatially structured. Industrial releases, traffic-related pollutants, air quality, drinking-water measures, and radiation-related monitoring are not randomly distributed across populations. Thyroid cancer incidence and stage patterns also vary across geography because of differences in screening, healthcare access, socioeconomic composition, race/ethnicity, registry coverage, and local diagnostic practices. Spatial analyses have shown that social and environmental determinants can jointly shape thyroid cancer patterns, with economic status, healthcare level, air pollution, green space, and other contextual factors showing spatial heterogeneity [16].

Socioeconomic context is particularly important in the air-pollution literature. Area-level deprivation, income, insurance access, screening intensity, and healthcare utilization may influence both measured pollutant exposure and thyroid cancer diagnosis. Crepeau et al. [17] reported a socioeconomic disparity in the association between PM2.5 day exposure and papillary thyroid cancer, underscoring the importance of evaluating environmental variables alongside demographic and socioeconomic predictors rather than treating environmental measures as independent of social context.

### 2.4. SEER-Based Research on Thyroid Cancer

SEER has been widely used to study thyroid cancer incidence, survival, treatment patterns, and population disparities because it provides large-scale, population-based cancer registry data with standardized information on patient demographics, tumor characteristics, stage, treatment, registry geography, and survival [22]. SEER-based studies have been especially important in documenting changes in thyroid cancer incidence over time. Davies and Welch [23] used SEER data to show that rising U.S. thyroid cancer incidence from 1973 to 2002 was largely driven by increased detection of small papillary cancers, while Lim et al. [2] examined longer-term incidence and mortality trends from 1974 to 2013. These studies helped establish SEER as a major resource for population-level thyroid cancer surveillance, but their primary focus was incidence and mortality rather than environmental context or advanced stage at diagnosis.

SEER has also supported studies of thyroid cancer prognosis and treatment. Adam et al. [24] used a large SEER cohort to evaluate extent of surgery and survival among patients with papillary thyroid cancer, demonstrating the value of SEER for studying clinical management and outcomes at population scale. More recently, Tatalovich et al. [18] described opportunities for linking SEER cancer patient data with environmental data sources, highlighting the potential for registry-based studies of exposure context. Building on this literature, the present study uses a large SEER thyroid cancer cohort with valid stage information to evaluate whether environmental exposures add information for advanced stage at diagnosis beyond demographic, socioeconomic, histologic, temporal, and registry-level predictors.

## 3. Methods

### 3.1. Study Design and Data Source

We conducted a retrospective, population-based registry-linked analysis of thyroid cancer stage at diagnosis using data from the Surveillance, Epidemiology, and End Results (SEER) Program of the National Cancer Institute. SEER is an authoritative U.S. cancer surveillance resource that collects and publishes cancer incidence and survival data from population-based cancer registries, including information on patient demographics, primary tumor site, tumor characteristics, stage at diagnosis, first course of treatment, and vital status follow-up [22].

Cancer case data were extracted through SEER*Stat from the SEER Research Plus Limited-Field Data, 22 Registries, November 2023 submission, which covered diagnosis years 2000–2021 [25]. Thyroid cancer cases were then selected from this SEER source file for the present analysis. Because the combined summary stage variable used to define the primary outcome was available for the relevant analytic period beginning in 2004, the effective stage-analysis period was 2004–2021. Each cancer record retained its SEER-assigned registry field; environmental linkage used the state-year structure available in the project dataset.

Environmental exposures were assembled from publicly available U.S. Environmental Protection Agency datasets. Air-pollutant and air-quality measures were derived from EPA AirData/Air Quality System data; heavy-metal and dioxin release proxies were derived from the EPA Toxics Release Inventory; and iodine-131 and beta-radiation proxies were derived from the EPA RadNet monitoring system [26,27,28]. Units for environmental exposures were retrieved from the original environmental data sources when available. For total heavy-metal release and total dioxin release, the available source files provided summarized release indicators but did not contain sufficiently documented measurement units. These variables were therefore interpreted as relative state-year release indicators rather than absolute exposure concentrations. SEER records were linked with environmental variables using the SEER-assigned registry/state-year information available in the analytic dataset. For example, records assigned to a given registry were linked to the corresponding state-year environmental measures used for that registry. Because individual residential histories and finer geographic identifiers were not available, these variables should be interpreted as registry/state-level environmental context rather than patient-level personal exposure measurements.

### 3.2. Cohort Construction

The analytic cohort was restricted to thyroid cancer records using Site Recode ICD-O-3/WHO 2008 == Thyroid. Records were then restricted to valid values of combined summary stage (2004+): in situ, localized, regional, or distant. Blank, unknown, or unstaged records were excluded from the primary analytic cohort because the binary stage outcome could not be assigned. In the final analytic cohort, 250,205 cases were localized, 104,617 were regional, 13,892 were distant, and 12 were in situ. The cohort selection process is summarized in Table 1. The SEER source file included 429,874 records, of which 426,131 were thyroid cancer site records; 3743 non-thyroid records were excluded. After restricting to thyroid cancer cases with valid combined summary stage, the final analytic cohort included 368,726 cases, including 250,217 early-stage cases and 118,509 advanced-stage cases, with advanced-stage disease representing 32.14% of the cohort.

Unknown race/ethnicity was retained as an explicit category in the primary cohort to avoid unnecessary case deletion. A known-race sensitivity cohort excluding records with unknown race/ethnicity was used to evaluate whether the primary findings were sensitive to race/ethnicity completeness.

Treatment and downstream variables were excluded from the primary predictor set because they may reflect clinical management after diagnosis or information closely related to disease stage. Excluded fields included treatment variables, surgery variables, lymph-node surgery scope, surgery/radiation sequence, year of death, and other redundant attributes.

### 3.3. Target Variable

The primary outcome was binary advanced stage at diagnosis. The combined summary stage (2004+) categories were mapped as follows: in situ and localized disease were coded as early stage (advanced_stage = 0), while regional and distant disease were coded as advanced stage (advanced_stage = 1). Records with blank, unknown, or unstaged summary stage were excluded from model fitting.

### 3.4. Predictor Variables and Model Feature Sets

Predictors were organized into sequential feature sets to evaluate whether environmental variables added information beyond standard demographic, socioeconomic, and tumor-related predictors. L1 included demographic and socioeconomic predictors: sex, age group, race/ethnicity, marital status, median household income category, rural–urban continuum code, and year of diagnosis. L2 added tumor-related predictors to L1, including a compact histology grouping derived from ICD-O-3 Hist/behav and laterality. Histology was collapsed to the 15 most common categories in the modeling cohort, with less common categories grouped as “Other.” L3 added 13 representative environmental exposure variables to L2. These variables included representative measures for total heavy-metal release, total dioxin release, PCBs, ozone, carbon monoxide, sulfur dioxide, nitrogen dioxide, iodine-131, beta radiation in drinking water, beta radiation from air-filter measurements, overall AQI, PM2.5 days, and PM10 days. Highly skewed nonnegative environmental variables were transformed using the natural logarithm of one plus the original value (log1p) before modeling, and missing environmental values were handled within the model pipelines rather than excluding otherwise eligible cases. L4 added SEER registry to L3 as a broad registry/geographic adjustment. To isolate environmental information after registry adjustment, we also fitted an explicit L2 + registry comparator and compared it with L2 + registry + environmental variables. Environmental variable units and missingness are summarized in Supplementary Table S3.

The intended prediction setting was a registry-based post-diagnosis and incremental information assessment using variables available after diagnostic work-up, rather than a pre-diagnostic clinical prediction model.

### 3.5. Machine Learning Model and Evaluation Metric Selections

Three model families were fitted using the same feature-set structure: regularized logistic regression, gradient boosting, and Extra Trees. Categorical variables were imputed with a missing category and encoded within model pipelines. Numeric variables were median-imputed; environmental variables were log1p-transformed when nonnegative and then standardized for logistic regression. Models used prespecified settings rather than extensive hyperparameter tuning so that results would remain transparent and comparable across feature sets.

The primary performance metric was test-set receiver operating characteristic area under the curve (ROC-AUC). We also evaluated precision–recall AUC, Brier score, calibration slope and intercept, and sensitivity/specificity at both a fixed 0.50 threshold and a train-derived Youden threshold, consistent with prediction-model evaluation principles [29,30]. Bootstrap resampling of test-set predictions was used to estimate 95% confidence intervals for ROC-AUC and paired bootstrap confidence intervals for AUC differences, including L3 versus L2 and L2 + registry + environmental variables versus L2 + registry. Additional performance metrics are provided in Supplementary Table S2.

### 3.6. Environmental Feature Importance

Environmental feature importance was evaluated using permutation importance. For registry-adjusted models, each representative environmental variable was permuted in the test data while all other variables were held fixed. Importance was measured as the decrease in test-set ROC-AUC after permutation. Because correlated ecological and geographic variables can make permutation rankings unstable, these values were interpreted as predictive importance measures rather than epidemiologic effect estimates or causal associations.

Permutation importance was computed on a stratified subset of up to 25,000 test rows for computational efficiency. Each environmental variable was permuted 10 times in the analysis, and mean, standard deviation, minimum, and maximum ROC-AUC decreases were reported.

### 3.7. Sensitivity Analyses

Sensitivity analyses were conducted to evaluate robustness and geographic generalizability. The L1–L4 logistic regression sequence was repeated in the known-race cohort, which excluded records with unknown race/ethnicity, and in two diagnosis-period subsets: 2004–2012 and 2013–2021. Gradient boosting sensitivity analyses repeated the same feature-set sequence in the known-race cohort and diagnosis-period subsets, while Extra Trees was retained as a primary model-family comparator in the primary-cohort analyses. To evaluate geographic transportability, registry-group holdout validation was performed with test registries unseen during training; because SEER registry itself could not be estimated for unseen registry levels, this validation compared L2 with L3.

## 4. Results

### 4.1. Cohort Characteristics and Descriptive Summaries

The final analytic cohort included 368,726 thyroid cancer records with valid combined summary stage. Early-stage disease included 250,205 localized and 12 in situ cases; advanced-stage disease included 104,617 regional and 13,892 distant cases, yielding 118,509 advanced-stage cases (32.14%). Selected baseline characteristics differed between early-stage and advanced-stage cases, although most differences were modest (Table 2). Full category-level baseline distributions are provided in Supplementary Table S1.

Race/ethnicity distributions were broadly similar between early-stage and advanced-stage cases, with White patients representing 81.6% of the overall cohort. Some Asian subgroup categories had higher advanced-stage proportions within level, while Black patients had a lower advanced-stage proportion within level. Marital status, income category, and rural–urban continuum code showed measurable but generally modest distributional differences by stage. Mean age at diagnosis was slightly lower among advanced-stage cases than early-stage cases (49.3 vs. 50.8 years), with a small standardized mean difference.

Environmental variables showed generally small descriptive differences by stage (Table 3). Because these variables were derived from multiple environmental data sources and measured on different scales, the absolute values in Table 3 should be interpreted as ecological state-year summaries rather than individual exposure values.

The largest descriptive differences were observed for ozone, nitrogen dioxide, sulfur dioxide, PM10 days, and iodine-131, but all were below traditional thresholds for large imbalance. This indicates that individual environmental variables were not strong univariate separators of early versus advanced stage.

### 4.2. Model Performance and Comparison

**Figure 1:**
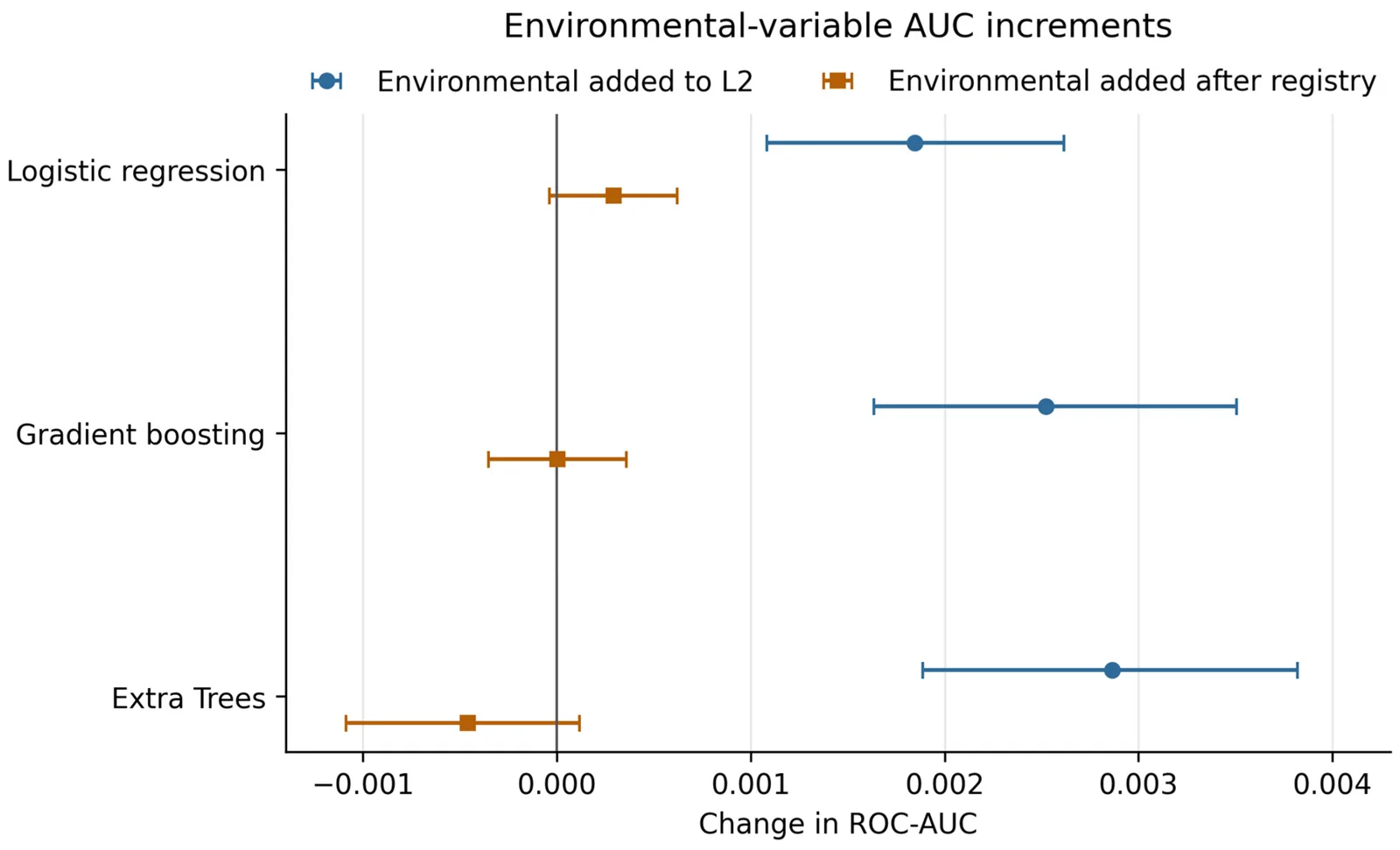
Environmental variable AUC increments before and after SEER registry adjustment.

Across all three model families, adding environmental variables to the demographic, socioeconomic, histologic, and laterality baseline produced small improvements in random-split test-set ROC-AUC (Table 4; Figure 1). In logistic regression, ROC-AUC increased from 0.6945 for L2 to 0.6963 for L3. In gradient boosting, ROC-AUC increased from 0.7003 to 0.7029. In Extra Trees, ROC-AUC increased from 0.6973 to 0.7001. Bootstrap confidence intervals for the paired L3 − L2 AUC differences were positive across the three model families.

However, the direct registry-adjusted comparison showed that much of the environmental information overlapped with SEER registry/geographic structure. After adding SEER registry to L2 first, the additional AUC change from adding environmental variables was +0.00029 for logistic regression, approximately 0.00000 for gradient boosting, and −0.00046 for Extra Trees. Thus, it is not that environmental variables provided a strong independent registry-adjusted improvement, but that they captured a small predictive signal that substantially overlapped with geographic registry context.

### 4.3. Registry-Adjusted Environmental Feature Importance

**Figure 2:**
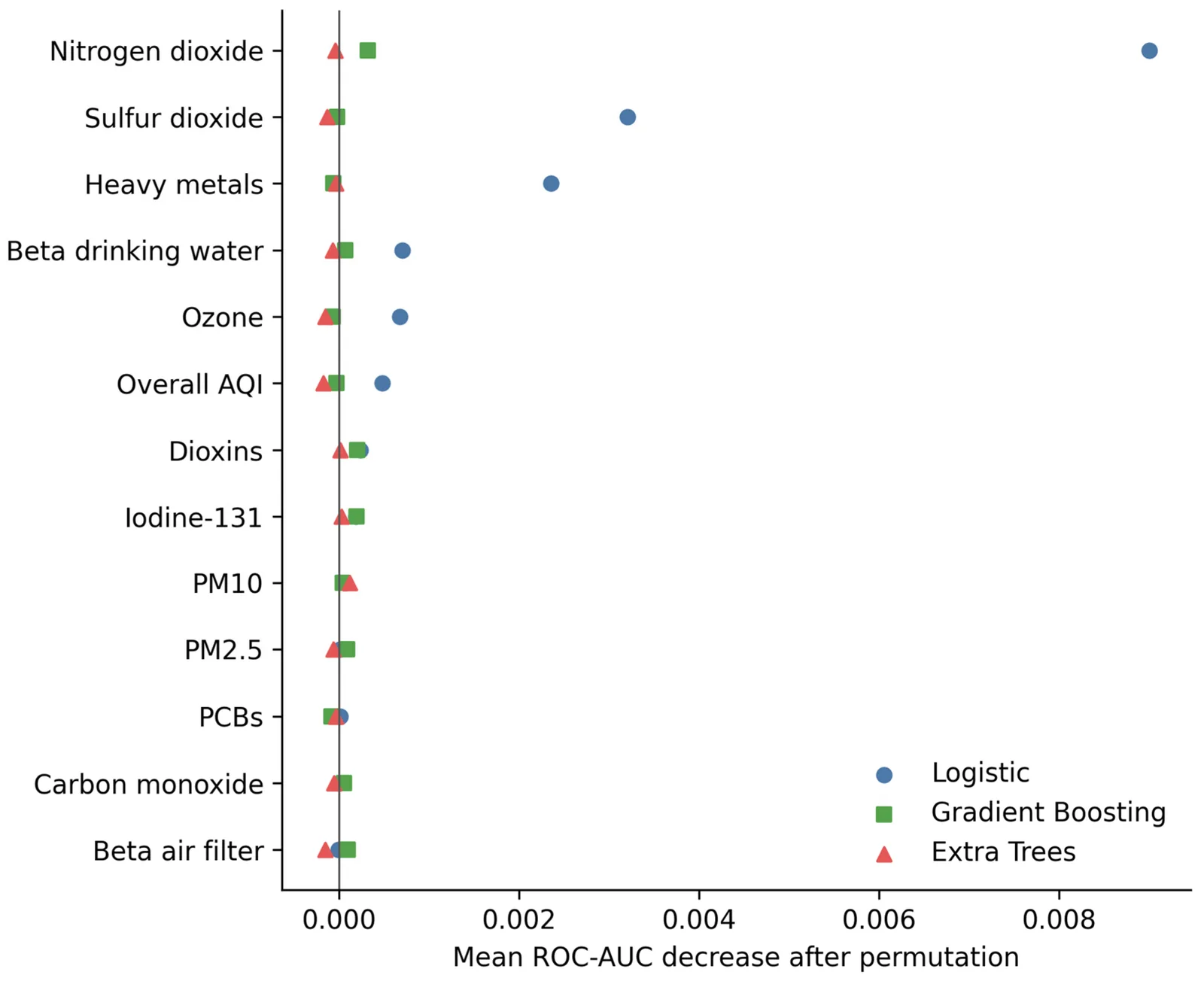
Registry-adjusted permutation importance for the 13 representative environmental variables. Values represent mean decreases in ROC-AUC after 10 test-set permutations.

Registry-adjusted environmental feature importance was evaluated using permutation importance in the model that included L2 predictors, SEER registry, and environmental variables. The measure is the mean decrease in test-set ROC-AUC after permuting each environmental variable while holding all other variables unchanged (Table 5; Figure 2).

In logistic regression, nitrogen dioxide had the largest registry-adjusted permutation importance among environmental variables, with a mean AUC decrease of 0.0090 across 10 permutation repeats. Sulfur dioxide and total heavy-metal release followed, with mean AUC decreases of 0.0032 and 0.0024, respectively. In gradient boosting and Extra Trees, individual environmental permutation decreases were much smaller and often close to zero. This difference across model families suggests that variable-specific rankings are sensitive to model form, feature correlation, and geographic structure.

Figure 2 displays the model-specific importance pattern. Overall, the permutation-importance results support cautious interpretation: some environmental variables, particularly nitrogen dioxide in logistic regression, were predictive within the fitted model, but this should not be interpreted as evidence that a single pollutant causally explains advanced stage at diagnosis.

### 4.4. Sensitivity Analyses

Sensitivity analyses qualified rather than simply confirmed the environmental signal (Table 6). In the patient-level random split and period/race sensitivity analyses, environmental variables generally improved AUC slightly relative to L2. However, the explicit post-registry comparison showed little additional gain after SEER registry was already included, and the registry-group holdout validation did not support improved geographic generalizability from adding the environmental feature set.

In registry-group holdout validation, test registries were unseen during training. Under this more conservative geographic validation design, L3 performed below L2 in logistic regression (0.6794 versus 0.6840), gradient boosting (0.6844 versus 0.6869), and Extra Trees (0.6839 versus 0.6850). These results suggest that environmental variables added limited predictive information across registry areas.

Taken together, the sensitivity analyses indicate that environmental variables can improve random patient-level prediction slightly, but that the environmental signal is geographically structured and should be interpreted cautiously.

## 5. Discussion

### 5.1. Principal Findings

In this large SEER-based thyroid cancer cohort, environmental exposure-context variables added small predictive increments for advanced stage at diagnosis in patient-level random-split models. This pattern was observed across regularized logistic regression, gradient boosting, and Extra Trees. The registry-adjusted analyses, however, showed that the environmental increment was much smaller once SEER registry was added before the environmental variables.

The magnitude of the random-split incremental AUC improvement was small, ranging from +0.0018 in logistic regression to +0.0029 in Extra Trees. Bootstrap analyses supported these L3 − L2 differences in the random split. By contrast, the environmental increment after registry adjustment was near zero in gradient boosting, slightly positive but not clearly robust in logistic regression, and slightly negative in Extra Trees. Registry-group holdout validation further suggested that environmental variables did not improve prediction for unseen registry areas.

### 5.2. Interpretation of Environmental Feature Importance

The environmental findings are most defensible as evidence of limited contextual predictive information rather than as evidence for one dominant exposure. The environmental variables were represented by 13 state-year five-year moving-average exposure-context indicators: total heavy-metal release, total dioxin release, PCBs, ozone, carbon monoxide, sulfur dioxide, nitrogen dioxide, iodine-131, beta radiation in drinking water, beta radiation from air-filter measurements, overall AQI, PM2.5 days, and PM10 days. These variables were modeled individually, not as a single composite environmental score.

Individual environmental rankings should be interpreted cautiously. In registry-adjusted permutation analyses, logistic regression assigned the greatest environmental importance to nitrogen dioxide, followed by sulfur dioxide and total heavy-metal release. Gradient boosting and Extra Trees produced much smaller and less stable registry-adjusted permutation decreases. The nitrogen dioxide result may reflect a combination of air-pollution context, urbanicity, socioeconomic patterning, screening or diagnostic access, and correlation with geography rather than an isolated pollutant-specific effect.

This interpretation is also supported by the descriptive analyses. The environmental variables showed only small standardized differences between early-stage and advanced-stage cases (Table 3). Thus, the environmental signal was not a simple univariate separation by a single exposure proxy, but a small multivariable predictive pattern embedded within geography and registry structure.

### 5.3. Relationship to Prior Literature

The findings are consistent with the previous literature in which environmental factors are biologically plausible but empirically heterogeneous. Prior studies have examined environmental exposures, air pollution, endocrine-disrupting chemicals, radiation-related factors, and social or geographic context in relation to thyroid cancer or thyroid-related outcomes [7,8,9,10,11,12,13,14]. However, conclusions differ across exposure classes, tumor subtypes, populations, outcome definitions, and analytic designs. Many studies focus on thyroid cancer incidence rather than advanced stage at diagnosis, and many examine single exposures or geographically restricted populations. This study contributes by shifting the question from whether one pollutant causes thyroid cancer to whether environmental exposure context adds information for clinically meaningful stage at diagnosis. The results support a modest environmental signal, but they also reinforce the complexity described in prior work.

### 5.4. Registry and Geographic Structure

SEER registry adjustment was central to the analysis because environmental variables linked at state-year levels may partly represent geography. The direct comparison of models with SEER registry alone versus models with both SEER registry and environmental variables showed that environmental variables added little additional AUC after registry adjustment. This suggests that much of the environmental information overlapped with registry and geographic structure. At the same time, individual environmental importance varied across model families, indicating that the environmental signal was geography-sensitive and model-dependent. The study therefore does not establish patient-level exposure effects or causal associations. Instead, it shows that state-year environmental context carried limited predictive information for advanced-stage thyroid cancer, much of which reflected broader geographic and registry structure.

## 6. Conclusions

This study used a large population-based SEER thyroid cancer cohort with valid summary stage to evaluate whether state-year environmental exposure-context variables contributed predictive information for advanced stage at diagnosis. Environmental variables slightly improved random-split prediction beyond demographic, socioeconomic, histology, and laterality predictors, but their additional contribution was minimal after the SEER registry had been included first. The study’s major strengths include its large population-based cohort, explicit stage-focused outcome, use of multiple model families, transparent feature-set comparisons, uncertainty analyses, and a registry-group holdout sensitivity analysis.

The study also has important limitations. Environmental variables were linked at the state-year level using five-year moving averages from the years preceding diagnosis and do not represent individual residential histories or personal exposure measurements. Some project-derived environmental streams did not have fully documented source units. Because the analysis was predictive and observational, feature importance should not be interpreted as a causal effect estimate. Residual confounding by screening, access to care, socioeconomic context, urbanicity, registry practices, and diagnostic changes over time may remain. Combined summary stage also spans a long study period, and stage comparability over time may be imperfect.

Future research should build on these findings using more detailed exposure linkage, including residential history and county-level or neighborhood-level environmental measures. Studies focused on causal inference could examine specific exposure classes with stronger designs, clearer latency assumptions, and external validation. For prediction, future work should evaluate whether environmental context improves transportable models across geographic regions, rather than relying only on patient-level random splits within the same registry structure.

## Figures and Tables

**Table 1:** Cohort construction.

Cohort Step	N	Note
Source data file	429,874	Original row count
Thyroid site records	426,131	Site Recode ICD-O-3/WHO 2008 == Thyroid
Non-thyroid records excluded	3743	NHL, myeloma, Hodgkin, miscellaneous, other myeloid/monocytic leukemia
Thyroid records with valid stage	368,726	Primary analytic cohort
Early stage	250,217	In situ or localized
Advanced stage	118,509	Regional or distant
Unknown race retained in primary cohort	2327	Known-race sensitivity cohort excludes these records
Known-race sensitivity cohort	366,399	After removing unknown race

**Table 2:** Selected Baseline characteristics by stage.

Characteristic	Overall	Early Stage	Advanced Stage	Advanced Rate
Age at diagnosis, years, median (IQR)	50 (39–62)	51 (40–62)	49 (36–62)	
Median household income, 2022 USD, median (IQR)	$77,500 ($67,500–$97,500)	$77,500 ($67,500–$92,500)	$77,500 ($67,500–$97,500)	
Female sex	276,222 (74.9%)	195,701 (78.2%)	80,521 (67.9%)	29.2%
Male sex	92,504 (25.1%)	54,516 (21.8%)	37,988 (32.1%)	41.1%
White race/ethnicity	300,936 (81.6%)	203,931 (81.5%)	97,005 (81.9%)	32.2%
Black race/ethnicity	26,171 (7.1%)	20,384 (8.1%)	5787 (4.9%)	22.1%
Other/Asian/Pacific Islander race/ethnicity	39,292 (10.7%)	24,136 (9.6%)	15,156 (12.8%)	38.6%
Unknown race/ethnicity	2327 (0.6%)	1766 (0.7%)	561 (0.5%)	24.1%
Married	170,767 (46.3%)	117,768 (47.1%)	52,999 (44.7%)	31.0%
Single, never married	65,249 (17.7%)	41,582 (16.6%)	23,667 (20.0%)	36.3%
Metropolitan county, ≥1 million population	244,275 (66.2%)	163,436 (65.3%)	80,839 (68.2%)	33.1%
Papillary adenocarcinoma, NOS	204,128 (55.4%)	126,222 (50.4%)	77,906 (65.7%)	38.2%

Note: The full category-level baseline table is provided as Supplementary Table S1.

**Table 3:** Environmental variables by stage.

Environmental Variable	Early-Stage Mean	Advanced-Stage Mean	Early-Stage Median (IQR)	Advanced-Stage Median (IQR)	SMD
Ozone (ppm)	0.0263	0.0266	0.027 (0.0245–0.0283)	0.0271 (0.0252–0.0283)	+0.089
Nitrogen dioxide (ppb)	13.9	13.6	13.8 (10.6–16.6)	13.4 (10.5–16.5)	−0.072
Sulfur dioxide (ppb)	1.78	1.69	1.16 (0.595–2.28)	1.09 (0.554–1.92)	−0.068
PM10 days (days)	99.9	110	38.8 (3.8–184)	51.8 (4.8–231)	+0.063
Iodine-131 (microBq/m^3^)	3.71 × 10^6^	3.85 × 10^6^	2.93 × 10^6^ (1.89 × 10^6^–5.02 × 10^6^)	3.08 × 10^6^ (1.89 × 10^6^–5.43 × 10^6^)	+0.052
PM2.5 days (days)	321	322	334 (301–362)	334 (301–362)	+0.030
Beta radiation in drinking water (microBq/m^3^)	1.19 × 10^8^	1.16 × 10^8^	9.02 × 10^7^ (5.72 × 10^7^–1.24 × 10^8^)	8.92 × 10^7^ (5.65 × 10^7^–1.23 × 10^8^)	−0.029
Overall AQI (AQI)	44.4	44.6	44 (40.5–48.2)	44.1 (40.5–51.1)	+0.028
Beta radiation from air-filter measurements (microBq/m^3^)	337	335	335 (283–366)	335 (285–364)	−0.028
PCBs (pg/L)	1.21 × 10^6^	1.22 × 10^6^	1.21 × 10^6^ (1.2 × 10^6^–1.24 × 10^6^)	1.22 × 10^6^ (1.2 × 10^6^–1.25 × 10^6^)	+0.026
Total heavy-metal release (source unit not documented)	1.34 × 10^7^	1.29 × 10^7^	7.89 × 10^6^ (1.01 × 10^6^–1.27 × 10^7^)	7.89 × 10^6^ (1.17 × 10^6^–1.26 × 10^7^)	+0.022
Carbon monoxide (ppm)	0.329	0.329	0.311 (0.27–0.379)	0.312 (0.27–0.369)	−0.006
Total dioxin release (source unit not documented)	4.66 × 10^3^	4.88 × 10^3^	110 (42.2–252)	110 (43.1–214)	−0.002

Note: Environmental variables are state-year exposure-context proxies represented as five-year moving averages from the years preceding diagnosis.

**Table 4:** Model performance and environmental increment before and after SEER registry adjustment.

Model Family	L2 AUC (95% CI)	L3 AUC (95% CI)	Delta L3 − L2 (95% CI)	L2 + Registry AUC (95% CI)	L2 + Registry + Env. AUC (95% CI)	Env. Delta After Registry (95% CI)
Logistic regression	0.6945 (0.6903–0.6982)	0.6963 (0.6922–0.7002)	0.0018 (0.0011–0.0026)	0.6970 (0.6927–0.7009)	0.6973 (0.6932–0.7013)	0.0003 (−0.0000–0.0006)
Gradient boosting	0.7003 (0.6962–0.7040)	0.7029 (0.6988–0.7069)	0.0025 (0.0016–0.0035)	0.7035 (0.6996–0.7074)	0.7035 (0.6997–0.7075)	0.0000 (−0.0004–0.0004)
Extra Trees	0.6973 (0.6932–0.7011)	0.7001 (0.6960–0.7041)	0.0029 (0.0019–0.0038)	0.7001 (0.6960–0.7039)	0.6997 (0.6955–0.7037)	–0.0005 (−0.0011–0.0001)

**Table 5:** Registry-adjusted environmental permutation importance across 10 repeats.

Environmental Variable	Logistic	Gradient Boosting	Extra Trees
Nitrogen dioxide	0.0090 ± 0.0006	0.0003 ± 0.0001	−0.0000 ± 0.0001
Sulfur dioxide	0.0032 ± 0.0005	−0.0000 ± 0.0001	−0.0001 ± 0.0001
Total heavy-metal release	0.0024 ± 0.0004	−0.0001 ± 0.0001	−0.0000 ± 0.0001
Beta radiation in drinking water	0.0007 ± 0.0001	0.0001 ± 0.0001	−0.0001 ± 0.0001
Ozone	0.0007 ± 0.0003	−0.0001 ± 0.0000	−0.0002 ± 0.0001
Overall AQI	0.0005 ± 0.0002	−0.0000 ± 0.0001	−0.0002 ± 0.0001
Total dioxin release	0.0002 ± 0.0002	0.0002 ± 0.0001	0.0000 ± 0.0001
Iodine-131	0.0002 ± 0.0001	0.0002 ± 0.0001	0.0000 ± 0.0001
PM10 days	0.0001 ± 0.0001	0.0000 ± 0.0001	0.0001 ± 0.0002
PM2.5 days	0.0000 ± 0.0000	0.0001 ± 0.0000	−0.0001 ± 0.0001
PCBs	0.0000 ± 0.0000	−0.0001 ± 0.0001	−0.0000 ± 0.0001
Carbon monoxide	0.0000 ± 0.0000	0.0001 ± 0.0001	−0.0001 ± 0.0001
Beta radiation from air-filter measurements	−0.0000 ± 0.0000	0.0001 ± 0.0001	−0.0002 ± 0.0001

**Table 6:** Registry-group holdout validation.

Model Family	L2 ROC-AUC	L3 ROC-AUC	Delta L3 − L2	L2 PR-AUC	L3 PR-AUC	L2/L3 Brier Score
Logistic regression	0.6840	0.6794	−0.0046	0.5176	0.5122	0.1999/0.2023
Gradient boosting	0.6869	0.6844	−0.0025	0.5244	0.5208	0.1990/0.2006
Extra Trees	0.6850	0.6839	−0.0011	0.5196	0.5187	0.2177/0.2156

## Data Availability

SEER cancer registry data are available from the National Cancer Institute SEER Program to authorized users under SEER data-use requirements. Environmental source data were obtained from publicly available U.S. Environmental Protection Agency resources, including the AirData/Air Quality System, Toxic Release Inventory data files, and RadNet. Derived analytic files and code may be made available by the corresponding author upon reasonable request, subject to SEER data-use restrictions.

## Supplementary Materials

The following supporting information can be downloaded at: https://www.mdpi.com/article/10.3390/cancers18162679/s1, Table S1: Full Baseline Characteristics by Stage; Table S2: Additional Model Performance Metrics; Table S3: Environmental Variable Units and Missingness.
