# Supplementary material for: Environmental Exposure Context and Advanced Stage at Diagnosis in Thyroid Cancer: A Population-Based SEER Analysis
Source: Cancers (Basel). 2026 Aug 19;18(16):2679. doi: 10.3390/cancers18162679 (PMC13510960; doi:10.3390/cancers18162679)
Supplement: Supplementary file 1 [file cancers-18-02679-s001.zip › cancers-4483173-supplementary.pdf]

Supplementary Tables

Table S1: Full Baseline Characteristics by Stage.

Characteristic	Level	Overall	Early stage	Advanced stage	Advanced rate within level, %	Balance statistic
Sex	Female	276,222 (74.9)	195,701 (78.2)	80,521 (67.9)	29.2	Cramer's V 0.111
Sex	Male	92,504 (25.1)	54,516 (21.8)	37,988 (32.1)	41.1	Cramer's V 0.111
Race/ethnicity	White	300,936 (81.6)	203,931 (81.5)	97,005 (81.9)	32.2	Cramer's V 0.077
Race/ethnicity	Black	26,171 (7.1)	20,384 (8.1)	5,787 (4.9)	22.1	Cramer's V 0.077
Race/ethnicity	Other Asian (1991+)	7,686 (2.1)	4,888 (2.0)	2,798 (2.4)	36.4	Cramer's V 0.077
Race/ethnicity	Chinese	6,909 (1.9)	4,152 (1.7)	2,757 (2.3)	39.9	Cramer's V 0.077
Race/ethnicity	Filipino	6,549 (1.8)	3,863 (1.5)	2,686 (2.3)	41.0	Cramer's V 0.077
Race/ethnicity	Other	3,203 (0.9)	2,176 (0.9)	1,027 (0.9)	32.1	Cramer's V 0.077
Race/ethnicity	Asian Indian (2010+)	2,503 (0.7)	1,551 (0.6)	952 (0.8)	38.0	Cramer's V 0.077
Race/ethnicity	Asian Indian or Pakistani, NOS (1988+)	2,443 (0.7)	1,466 (0.6)	977 (0.8)	40.0	Cramer's V 0.077
Marital status at diagnosis	Divorced	19,741 (5.4)	14,004 (5.6)	5,737 (4.8)	29.1	Cramer's V 0.052
Marital status at diagnosis	Married (including common law)	170,767 (46.3)	117,768 (47.1)	52,999 (44.7)	31.0	Cramer's V 0.052
Marital status at diagnosis	Not available (IL or TX)	80,914 (21.9)	54,700 (21.9)	26,214 (22.1)	32.4	Cramer's V 0.052
Marital status at diagnosis	Separated	2,881 (0.8)	1,991 (0.8)	890 (0.8)	30.9	Cramer's V 0.052
Marital status at diagnosis	Single (never married)	65,249 (17.7)	41,582 (16.6)	23,667 (20.0)	36.3	Cramer's V 0.052
Marital status at diagnosis	Unknown	13,318 (3.6)	9,844 (3.9)	3,474 (2.9)	26.1	Cramer's V 0.052
Marital status at diagnosis	Unmarried or Domestic Partner	1,064 (0.3)	650 (0.3)	414 (0.3)	38.9	Cramer's V 0.052
Marital status at diagnosis	Widowed	14,792 (4.0)	9,678 (3.9)	5,114 (4.3)	34.6	Cramer's V 0.052
Median household income inflation adj to 2022	$100,000 - $109,999	35,571 (9.6)	24,132 (9.6)	11,439 (9.7)	32.2	Cramer's V 0.038
Median household income inflation adj to 2022	$110,000 - $119,999	18,319 (5.0)	12,006 (4.8)	6,313 (5.3)	34.5	Cramer's V 0.038
Median household income inflation adj to 2022	$120,000+	16,895 (4.6)	10,895 (4.4)	6,000 (5.1)	35.5	Cramer's V 0.038
Median household income inflation adj to 2022	$40,000 - $44,999	6,357 (1.7)	4,622 (1.8)	1,735 (1.5)	27.3	Cramer's V 0.038
Median household income inflation adj to 2022	$45,000 - $49,999	9,534 (2.6)	6,779 (2.7)	2,755 (2.3)	28.9	Cramer's V 0.038
Median household income inflation adj to 2022	$50,000 - $54,999	11,664 (3.2)	8,262 (3.3)	3,402 (2.9)	29.2	Cramer's V 0.038
Median household income inflation adj to 2022	$55,000 - $59,999	21,501 (5.8)	15,060 (6.0)	6,441 (5.4)	30.0	Cramer's V 0.038
Median household income inflation	$60,000 - $64,999	33,326 (9.0)	23,360 (9.3)	9,966 (8.4)	29.9	Cramer's V 0.038

adj to 2022						
Median household income inflation adj to 2022	$65,000 - $69,999	37,026 (10.0)	25,174 (10.1)	11,852 (10.0)	32.0	Cramer's V 0.038
Median household income inflation adj to 2022	$70,000 - $74,999	52,882 (14.3)	35,203 (14.1)	17,679 (14.9)	33.4	Cramer's V 0.038
Median household income inflation adj to 2022	$75,000 - $79,999	30,141 (8.2)	20,388 (8.1)	9,753 (8.2)	32.4	Cramer's V 0.038
Median household income inflation adj to 2022	$80,000 - $84,999	30,050 (8.2)	19,973 (8.0)	10,077 (8.5)	33.5	Cramer's V 0.038
Median household income inflation adj to 2022	$85,000 - $89,999	18,840 (5.1)	12,956 (5.2)	5,884 (5.0)	31.2	Cramer's V 0.038
Median household income inflation adj to 2022	$90,000 - $94,999	22,536 (6.1)	15,188 (6.1)	7,348 (6.2)	32.6	Cramer's V 0.038
Median household income inflation adj to 2022	$95,000 - $99,999	21,407 (5.8)	14,281 (5.7)	7,126 (6.0)	33.3	Cramer's V 0.038
Median household income inflation adj to 2022	< $40,000	2,654 (0.7)	1,923 (0.8)	731 (0.6)	27.5	Cramer's V 0.038
Median household income inflation adj to 2022	Unknown/missing/no match/Not 1990-2022	23 (0.0)	15 (0.0)	8 (0.0)	34.8	Cramer's V 0.038
Rural-urban continuum Code	Counties in metropolitan areas more than 1 million population	244,275 (66.2)	163,436 (65.3)	80,839 (68.2)	33.1	Cramer's V 0.033
Rural-urban continuum Code	Counties in metropolitan areas of 250,000 to 1 million population	67,778 (18.4)	46,523 (18.6)	21,255 (17.9)	31.4	Cramer's V 0.033
Rural-urban continuum Code	Counties in metropolitan areas of less than 250,000 population	23,998 (6.5)	17,093 (6.8)	6,905 (5.8)	28.8	Cramer's V 0.033
Rural-urban continuum Code	Nonmetropolitan counties adjacent to a metropolitan area	20,632 (5.6)	14,703 (5.9)	5,929 (5.0)	28.7	Cramer's V 0.033
Rural-urban continuum Code	Nonmetropolitan counties not adjacent to a metropolitan area	11,809 (3.2)	8,316 (3.3)	3,493 (2.9)	29.6	Cramer's V 0.033
Rural-urban continuum Code	Unknown/missing/no match (Alaska or Hawaii - Entire State)	211 (0.1)	131 (0.1)	80 (0.1)	37.9	Cramer's V 0.033
Age at diagnosis	Mean (SD); median (IQR)	50.3 (15.9); 50 (39-62)	50.8 (15.1); 51 (40-62)	49.3 (17.4); 49 (36-62)		SMD -0.089
Median household income midpoint	Mean (SD); median (IQR)	80,271 (19,848); 77,500 (67,500-97,500)	79,879 (19,862); 77,500 (67,500-92,500)	81,099 (19,793); 77,500 (67,500-97,500)		SMD +0.061

Table S2: Additional Model Performance Metrics.

**Model Family**	**Model**	**ROC-AUC**	**PR-AUC**	**Brier Score**	**Calibration Slope**	**Sensitivity**	**Specificity**	**Youden Sensitivity**
Logistic	L2	0.6945	0.5226	0.1948	1.0113	0.1995	0.9418	0.6209
Logistic	L3	0.6963	0.5241	0.1944	1.0094	0.2057	0.9391	0.6337
Logistic	L2_registry	0.6970	0.5245	0.1942	1.0095	0.2057	0.9392	0.6073
Logistic	L2_registry_environment	0.6973	0.5253	0.1941	1.0091	0.2072	0.9384	0.6480

Gradient Boosting	L2	0.7003	0.5318	0.1932	1.0456	0.2238	0.9339	0.6327
Gradient Boosting	L3	0.7029	0.5344	0.1926	1.0536	0.2296	0.9323	0.6560
Gradient Boosting	L2_registry	0.7035	0.5347	0.1925	1.0355	0.2365	0.9299	0.6824
Gradient Boosting	L2_registry_environment	0.7035	0.5347	0.1925	1.0373	0.2357	0.9294	0.6657
Extra Trees	L2	0.6973	0.5267	0.2195	1.1399	0.6378	0.6425	0.6579
Extra Trees	L3	0.7001	0.5284	0.2186	1.1920	0.6436	0.6408	0.6576
Extra Trees	L2_registry	0.7001	0.5276	0.2188	1.1640	0.6507	0.6331	0.6588
Extra Trees	L2_registry_environment	0.6997	0.5270	0.2188	1.1840	0.6493	0.6344	0.6589

Table S3: Environmental Variable Units and Missingness.

**Environmental variable**	**Unit or scale**	**Missing, n**	**Missing, %**
Total heavy-metal release	source unit not documented	211	0.057
Total dioxin release	source unit not documented	13462	3.651
Polychlorinated biphenyls (PCBs)	pg/L	211	0.057
Ozone	ppm	211	0.057
Carbon monoxide	ppm	211	0.057
Sulfur dioxide	ppb	211	0.057
Nitrogen dioxide	ppb	211	0.057
Iodine-131	microBq/m3	37724	10.231
Overall beta radiation in drinking water	microBq/m3	36423	9.878
Overall beta radiation in air-filter samples	microBq/m3	11456	3.107
Median Air Quality Index	AQI	211	0.057
PM2.5 days	days	211	0.057
PM10 days	days	211	0.057
